# Effects of Vacuum and Modified Atmosphere Packaging on the Quality and Shelf-Life of Gray Triggerfish (*Balistes capriscus*) Fillets

**DOI:** 10.3390/foods10020250

**Published:** 2021-01-26

**Authors:** Eduardo Esteves, Luís Guerra, Jaime Aníbal

**Affiliations:** 1 Departamento de Engenharia Alimentar, Instituto Superior de Engenharia, Universidade do Algarve—Campus da Penha, 8005-139 Faro, Portugal; luis.vhs.guerra@gmail.com (L.G.); janibal@ualg.pt (J.A.); 2 CCMAR-Centro de Ciências do Mar, Universidade do Algarve—Campus de Gambelas, 8005-139 Faro, Portugal; 3 CIMA-Centro de Investigação Marinha e Ambiental, Universidade do Algarve—Campus de Gambelas, 8005-139 Faro, Portugal

**Keywords:** triggerfish, quality, vacuum, modified atmosphere package, shelf-life

## Abstract

Seafood products are perceived as healthy foods. However, several species of seafood are still not fully utilized for different reasons or can be valued outside the original locale, if issues with the short shelf-life and/or the preparation/presentation form are overcome, e.g., gray triggerfish, *Balistes capriscus*. Consumed mostly fresh, its flesh is of excellent quality. We studied the effect of different types of packaging (in air (AIR), vacuum (VP), and modified atmosphere (MAP)) on physicochemical (color and texture, pH, and total volatile basic nitrogen), microbiological (total viable count, psychrotrophic, sulphide-reducing bacteria, and acid-lactic bacteria), and sensory qualities, and shelf-life of gray triggerfish fillets stored at refrigeration temperature for 15 days. The samples were analyzed on days 0 (fresh fish), 5, 10 (8 and 12 for sensory analysis), and 15 after filleting and packaging. During the trial, fillets became lighter (increased L*) and yellower (b* >> 0) with time of storage. Distinct patterns were observed for pH among treatments. Unexpectedly, the increasing trend observed in the texturometer-derived hardness of VP and MAP fillets, contrasted with the sensory assessment, wherein panelists perceived a clear softening of fillets. VP delayed and MAP inhibited the increase in TVB-N contents of fillets compared to fillets packed in AIR. Total viable count and psychrothropic bacteria of fillets in AIR exceeded the conventional limit of 7 log(CFU/g) on day 10, while in fillets packed in VP and MAP, their abundance remained below that limit during the trial. The organoleptic attributes of fillets perceived by a sensory panel changed significantly in all treatments during the storage trial. Willingness to consume the fillets decreased constantly in AIR and MAP, but not in VP fillets. Considering primarily sensory, but also biochemical and microbiological parameters, namely panelists’ rejection, total volatile basic nitrogen content, and total viable count and psychrotrophic bacteria abundance, the shelf-life of fillets packed in air was eight days. Vacuum and modified atmosphere packaging extended the shelf-life to 15 and 12 days, and thus can add value to this product. Future research regarding the VP and MAP of gray triggerfish fillets could involve the optimization of mixtures of gases use and/or the application of combined processes.

## 1. Introduction

Consumers regard seafood and seafood products as healthy food products [1,2,3]. Notwithstanding, several species of seafood are still not fully utilized in many regions for different kinds of reasons, such as social/religious constraints and/or unfavorable appearance. Other species that are locally recognized can be valued outside the original locale, but issues with the short shelf-life relative to the duration of distribution and/or the preparation/presentation form need to be overcome, e.g., the gray triggerfish.

Gray triggerfish, *Balistes capriscus*, is widespread in the Atlantic Ocean (incl. the Gulf of Mexico) and the Mediterranean Sea. Consumed mostly fresh, smoked, and dried salted, its flesh is of excellent quality [4]. Until recently, it was not considered a desirable catch by most fishers. However, it is significant in the Gulf of Mexico and Brazil, and in subtropical regions such as in the Gulf of Guinea [5,6,7,8].

Fish and seafood are important sources of protein, polyunsaturated fatty acids (PUFA), vitamins, and minerals, thus contributing to human health. However, the contents in non-protein nitrogenous compounds and lipids, the weak connective tissue, and the high moisture content, make fish and fishery products susceptible to post-mortem deterioration. This is due to autolytic, microbiological, and chemical phenomena [9,10].

The nutritional composition of gray triggerfish muscle [11,12] is comparable to other lean fishes. Besides nutritional composition, size, contaminating microbiota, season and/or fishing gear used and manipulation during transportation, storage and/or processing contribute to deterioration and freshness/quality loss after catch. To keep species’ organoleptic conditions, it is necessary to store them properly [13]. Chilling storage of fish in melting ice, refrigeration, or freezing are widely used [14]. Pacheco-Aguilar et al. [12] found that finescale triggerfish *B. polylepsis* has excellent shelf-life on ice (at 0–3 °C), exceeding 20 days.

Testing “light”, minimal preservation, or other proper packaging methods other than icing or chilling is timely to prolong the shelf-life [15,16]. Modified atmospheres have been used in seafood for some time, and are the subject of literature reviews since the 1990s [17,18,19,20,21]. In modified atmosphere packaging (MAP), a gas mixture different from atmospheric air substitutes the surrounding atmosphere of the seafood product. It delays microbial growth, retards the trimethylamine (TMA) and total volatile basic nitrogen (TVB-N) formation, and partly maintains desirable sensorial properties. In contrast, MAP has added costs, requires temperature control, and involves special equipment [17,18,19,20,21]. Vacuum packaging (VP) can be understood as a particular case of MAP wherein the maximum amount of air inside a packaged seafood product is removed. VP combined with chilled storage, extends the shelf-life of seafood products, by limiting the availability of O_2_ necessary for the growth of aerobic bacteria. Moreover, VP involves a suitable moisture and gas permeability, and allows the assembly and protection from the contamination with undesirable substances from outer environment [17]. Both VP and MAP increase shelf-life. To our knowledge, neither VP or MAP have yet been applied and studied in the case of triggerfish fillets.

This study contributes to finding viable and sustainable alternatives for under- or not-valued species, non-targeted (by-catch) species, or undersized (targeted) specimens landed, e.g., in the context of EU’s discard ban [22], without creating a market that promotes its overfishing while going beyond the (more) “traditional” alternatives, viz. fish oil and fish feed, and envisaging human feeding.

The objectives of this study were to experimentally assess the changes in several freshness/quality indices (physicochemical, microbiological, and sensory parameters) and to empirically estimate the shelf-life of gray triggerfish fillets packed in air, vacuum, and modified atmosphere during storage at refrigeration temperatures (4 ± 1 °C). It was hypothesized that VP and/or MAP could contribute to keep the freshness/quality and prolong the shelf-life of gray triggerfish fillets.

## 2. Materials and Methods

### 2.1. Materials

Fresh specimens (*n* = 18, 337.7 ± 48.0 g) of gray triggerfish, *Balistes capriscus*, caught overnight were purchased in the local fish market in Faro (South Portugal), transported in insulated cooler boxes in scaled ice to the laboratory at the University of Algarve (Faro, Portugal). The fish were washed with tap water and filleted under hygienic conditions. The average (±SD) weight of fillets was 69.7 ± 11.6 g.

### 2.2. Packaging and Storage Conditions

Fillets were immediately and individually packed in Combitherm^®^ XX (Wolff Walsrode AG, Germany) bags (200 × 200 mm) under atmospheric air (AR, sealing only), vacuum (VP, at ca. 380 mm Hg), and modified atmosphere (MAP, 30% N_2_ + 40% CO_2_ + 30% O_2_) using a multipack vacuum packaging system (Interdibipack S.p.a., Italy). The packaging film was coextruded laminate composed of an exterior cast polyamide (PA) layer, a coextruded interior barrier layer containing ethylene vinyl alcohol (EVOH), and a polyethylene (PE) sealing layer. For this film, the oxygen transmission rate (OTR) is 0.5 cm^3^ m^−2^ d^−1^ bar at 23 °C and 85% relative humidity. Storage trials were carried out for 15 days at 4 ± 1 °C.

### 2.3. Physicochemical Analyses

Color measurements were carried out directly on fresh and packed/chilled stored samples using a tristimulus colorimeter (model DR LANGE, Spectro-color, Spain) and examined according to the CIE Lab color scale (CIE L*a*b*), where L* refers to lightness (0 is black and 100 is white), a* indicates greenness (a < 0) or redness (a > 0), and b* measures blueness (b < 0) or yellowness (b > 0) of samples. The colorimeter was calibrated using black and white control tiles according to manufacturer instructions. Chroma (C) and hue angle (h) were also determined as $C=\sqrt{a^{*2}+b^{*2}}\;$and $h=\tan^{-1}\left(b^{*}/a^{*}\right)$, respectively. As a summary measure, total color change (denoted ΔE) was calculated in accordance with $\Delta E^{*}=\;\sqrt{{\left(\Delta L^{*}\right)}^{2}\;+\;{\left(\Delta a^{*}\right)}^{2}\;+\;{\left(\Delta b^{*}\right)}^{2}\;}$, where for example $\Delta L^{*}=\left(L_{t}^{*}-L_{0}^{*}\right)$ and L* refers to lightness at time *t* (L*t) and time 0 (L*0). pH was determined directly in fish flesh using a digital meter (model Glp 21, Crison, Spain) that was calibrated routinely with standard solutions having pH 4 and 7 according to manufacturer instructions. Chemical spoilage was assessed using TVB-N according to the microdiffusion method described by Conway and Byrne as per Portuguese standard NP 2930 and Regulation CE 2074/2005 [23,24]. The muscle content in TVB-N was expressed as mg N/100 g. Hardness, i.e., the maximum force required to attain a deformation of the sample’s surface [25], was determined via a compression test. This test was carried out using a texturometer (LFRA Texture Analyzer, Brookfield Engineering Labs Inc., USA) equipped with a 12.7 mm-diameter stainless steel spherical probe which approached the sample at 1 mm s^−1^ and compressed 5 mm into the fillet. Measurements (in kgf, where 1 kgf = 9.806 N) were analyzed using TexturePro Lite v1.1 software (Brookfield Engineering Labs Inc., USA). Hardness (in N) was calculated as the peak load of the first compression cycle.

### 2.4. Microbiological Analyses

Samples (10 g) of fillets were aseptically placed into sterile Stomacher^®^ bags containing 90 mL of peptone water with NaCl (0.85% w/v) (Merck, Darmstadt, Germany) and homogenized for 2 min (Stomacher^®^ 400, Seward Ltd., London, UK). Aliquots of 1 mL were poured in Petri dishes according to serial decimal dilutions before addition of appropriate media. For the enumeration of mesophilic aerobic and psychrotrophic bacteria, PCA (Scharlau 01-161, Germany) was incubated at 30 °C for 2 days [26] and at 6.5 °C for 10 days [27], respectively. Lactic acid bacteria (LAB) were enumerated after inoculation of 1 mL aliquots into 10 mL of MRS agar (Scharlau 01-135, Germany), respectively. After settling, a 10 mL overlay of molten media was added, and plates were incubated 30 °C for 48 h [28]. For hydrogen sulphide (H_2_S)-reducing bacteria, Iron Agar (IA) was prepared and used according to International standard ISO 15213 [29]. Specifically, a thin overlay of IA was poured on top of the IA to avoid the fading of the black colonies due to oxidation of iron sulphide (FeS). Petri dishes were then incubated at 25 °C for 72 h, and black colonies were counted as H_2_S-reducing bacteria. All plates were examined visually for typical colony types and morphological characteristics associated with each medium. Microbiological data, i.e., number of colony-forming units per unit mass, were log-transformed prior to analysis, log(CFU/g).

### 2.5. Sensory Analysis

Sensory evaluation of fillets was conducted on days 0, 1, 5, 8, 12, and 15 during the storage trial using a panel of 16 individuals (18–65 years-old, 69% were women) co-opted from the faculty, staff, and graduate students of the Department of Food Engineering (ISE, University of Algarve), that are regular consumers of seafood (43% consume seafood >3–4 times/week) and experienced in the sensory assessment of seafood products, and were selected at the end of a training period that encompassed several sessions.

Each panelist assessed five sensory attributes: Appearance/color, odor, elasticity, firmness, and overall assessment using a 5-point scale (Table 1) that was adapted from Meilgaard et al. [30]. The panelists were also asked if at that time they would consume the samples or not. The sessions were carried out under standardized laboratory conditions that follow Portuguese/international standard NP EN ISO 8589 [31].

### 2.6. Experimental Design and Statistical Analysis

The analyses described above were carried out on days 0, 1, 5, 10 (8 and 12 in the case of pH and sensory analysis), and 15 after filleting/packaging. On each occasion, *n* = 2 fillets were sampled. Several measurements were made on each fillet and averaged: Three for pH, five for color, two for TVB-N, and six for hardness. The average values per fillet were the data used in the statistical analysis. Two-way ANOVA with factors storage time (days) and package type (AIR, VP, and MAP) was carried out for each of the quality parameters analyzed. In the case of physicochemical and microbiological parameters, due to non-homogenous variances (Levene tests, *p* < 0.05), the ANOVA model used heteroscedastic-corrected covariance matrices as implemented in the R package car [32]. All statistical procedures were carried out at the 0.05 level of significance and using R statistical software [33]. Results are expressed as estimates/means ± standard deviation (or standard error, as appropriate).

## 3. Results and Discussion

We assessed the effect of packaging gray triggerfish fillets in air (AIR), in vacuum (VP), and in modified atmosphere (MAP) on various quality parameters, during a 15-days storage trial at refrigerating temperature.

### 3.1. Physicochemical Parameters

**Figure 1:**
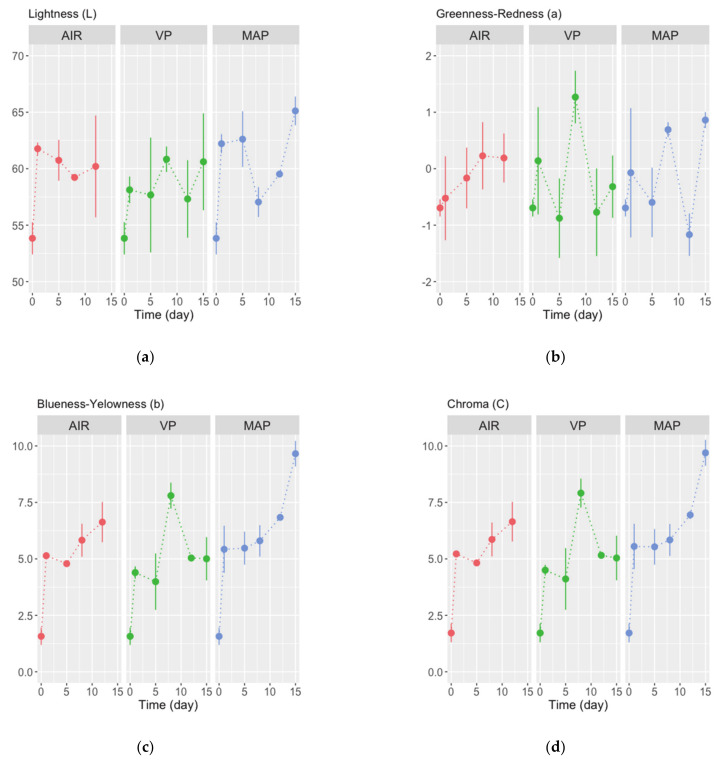

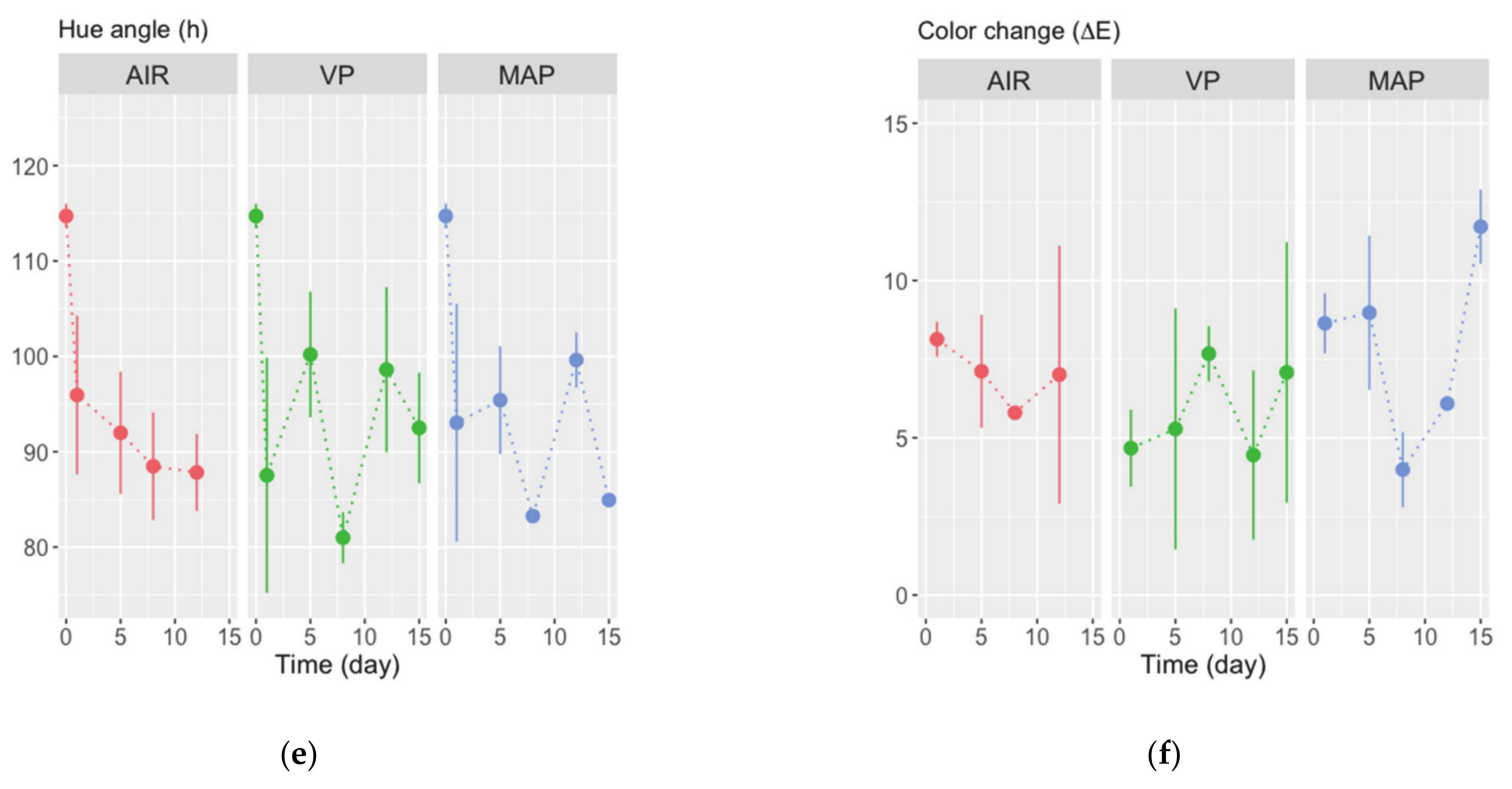
Changes in color parameters, (**a**) L*, (**b**) a*, (**c**) b*, (**d**) chroma, (**e**) hue angle, and (**f**) ΔE, measured in fillets of triggerfish packed in air (AIR), vacuum (VP), and modified atmosphere (MAP) stored refrigerated for 15 days. Values are means of *n* = 2 fillets and whiskers represent the standard-error (SE).

During the storage trials, the color of triggerfish fillets changed appreciably (Figure 1). There were significant differences (ANOVA, *p* < 0.05) among days of storage in color parameters L* (lightness), a* (redness–greenness), and b* (blueness–yellowness) (Table 2). With time of storage, fillets’ lightness increased, and they became yellower (b* >> 0). Alterations in heme proteins [34] and/or drip (loss) channels [35] have been suggested to increase L* in fish fillets. On the other hand, lipid oxidation can be a reason for the increase b*. The differences in b* affected the observed significant (ANOVA, *p* < 0.05) changes in chroma and the hue angle (h*) (hue is what we usually refer to as color). There were no significant differences in (total) color changes (ΔE) between sampling times and/or packaging (ANOVA, *p* > 0.05). However, ΔE values were always higher than 4 (8.1 > ΔE > 5.8 in AIR, 7.7 > ΔE > 4.5 in VP, and 11.7 > ΔE > 4.0 in MAP). This indicates that after only one day of storage, the differences are clearly distinct and/or perceptible at a glance [36,37,38] and remained consistent during the trial. In fact, changes in appearance/color were noticed by panelists (see below).

**Figure 2:**
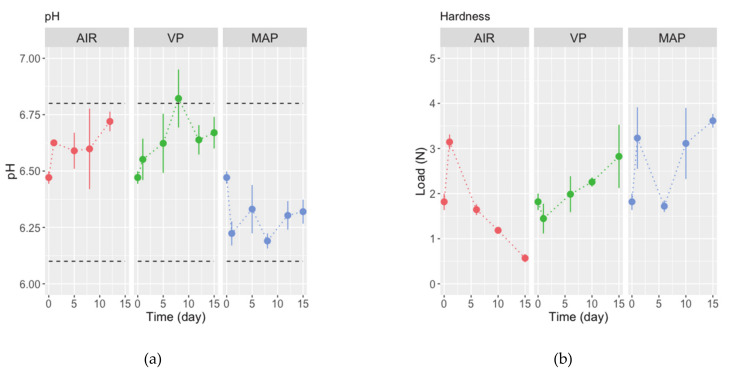

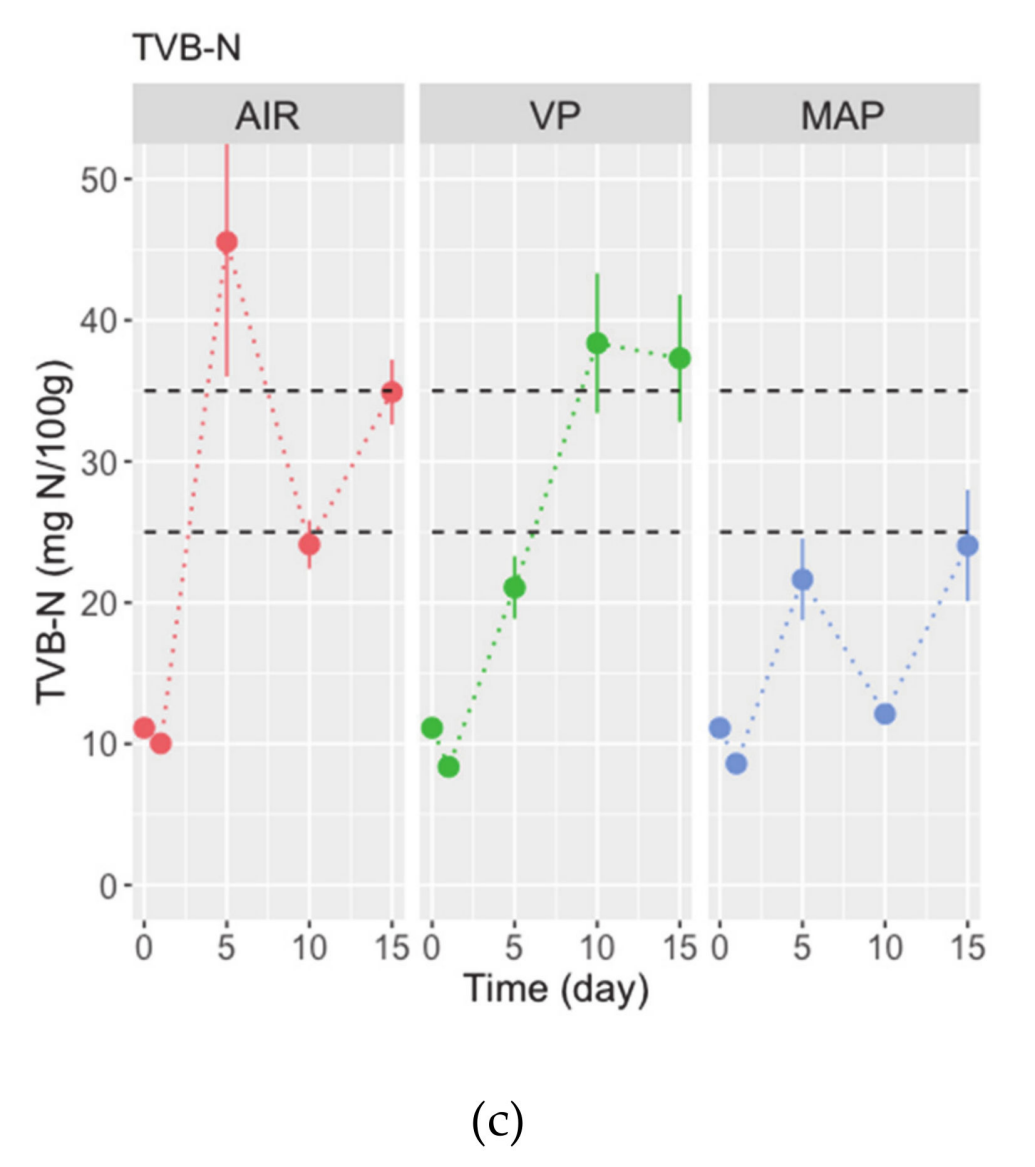
Changes in (**a**) pH, (**b**) hardness, and (**c**) TVB-N content in fillets of triggerfish packed in air (AIR), vacuum (VP), and modified atmosphere (MAP), and stored refrigerated for 15 days. Values are means of n = 2 fillets, and whiskers represent the standard-error (SE). Horizontal dashed lines in (**a**) represent the range of pH values referred for fresh fish [39], and in (**c**) represent the range of TVB-N values stipulated in EU Regulations [24,45].

The pH of fresh triggerfish fillets was 6.47 ± 0.06 (±SD). This is in line with values published for fresh fish by Howgate [39], pH 6.1–6.8, and for finescale triggerfish *B. polylepis* by Pacheco-Aguillar et al. [12], 6.38 ± 0.02. In fillets packed in air, the pH increased regularly to 6.72 ± 0.06 after 12 days (Figure 2a). Likewise, the pH of VP fillets increased to 6.82 ± 0.18 after 8 days, but then decreased to 6.67 ± 0.10. In contrast, fillets in MAP showed an initial sharp decrease (to pH < 6.25); afterwards, pH values fluctuated around 6.19–6.32. These distinct patterns are reflected in the significance of the interaction term in ANOVA (*p* = 0.0108; Table 2).

The increment observed in pH of fillets in AIR and VP could be due to postmortem microorganisms’ metabolism. This produces basic compounds from nitrogen deamination (also reflected by the increase in levels of TVB-N) [40]. Interestingly, in fillets of triggerfish *B. polylepis* stored chilled at 0–3 °C for 20 days, Pacheco-Aguillar et al. [12] found that pH increased slightly. Diverse dynamics in pH have been found for other lean fish species’ fillets in AIR or VP. For example, Massa et al. [41] observed that the pH of flounder stored refrigerated (~in AIR) kept constant for 6 days and then increased (until day 10). In barramundi, the pH of fillets stored at 4 °C for 21 days showed a similar trend [42]. The pH in meagre fillets stored in AIR and VP at 4 °C increased initially and then decrease to a level close to initial values [13]. A similar pattern was observed in rainbow trout fillets stored at 1 ± 1 °C [43]. Sáez et al. [44] found that pH values in meagre fillets declined when packed in AIR, VP, or MAP and stored at ca. 4 °C. The early sharp decrease observed in the pH values of fillets in MAP, might result from the impregnation of mixtures gases, mainly CO_2_, that acidified the internal medium when converted to carbonic acid [20,43]. pH changes in fish muscle are arguably a reliable indicator of quality per se, but herein they were partially related to changes observed in TVB-N content and the microbiota (see below).

Changes in fillets’ hardness are depicted in Figure 2b. After an initial, steep increase in hardness (peak load) from 1.82 ± 0.32 N to 3.14 ± 0.96 N, fillets in AIR became softer with storage time (0.57 ± 0.13 N on day 15). In contrast, the hardness of fillets in VP declined during the first 24 h of storage (to 1.45 ± 0.47 N) and increased steadily until day 15 (when the peak load was 2.82 ± 0.99 N). Distinctively, the fillets in MAP showed a fluctuating pattern in hardness between 1.72 ± 0.18 N (day 6) and 3.61 ± 0.22 N (day 15). The significance of terms in ANOVA (*p* < 0.0001; Table 2) reflects the changes described above.

The initial hardness of triggerfish fillets, ca. 1.8 N, is higher than the “firmness” of the tetraodontid obscure pufferfish [46], ca. 0.9 N, close to values found for meagre fillets by Genç et al. [13], ca. 2.4 N, but considerably lower than those found by Sáez et al. [44] also for meagre fillets, ca. 30 N. Besides inter-specific differences in morphology and the interaction between myofibrillar proteins and water during storage [47], numerous factors affect texture measurements in fish and fish products [48]. Differences in equipment and procedures, as well as the size of fillets could have influenced the texture determinations. Seafood deterioration is commonly associated with softening of the tissues because of proteolysis and pH levels. For example, decreasing hardness values were observed in cultured and wild sea bream fillets [49,50]. The trend observed in triggerfish fillets in AIR is seemingly typical during chilled storage [47,51]. Unexpectedly, the increasing trend observed in the hardness of VP and MAP fillets in this study can result from pH variations and bacterial activity that affect protein conformation, water-holding capacity (WHC), and yield higher hardness values [47]. These results of instrumental assessment of texture contrast with the sensory assessment (see below), wherein panelists perceived a clear softening of fillets during the storage trial.

When looking at the TVB-N content, a regulated quality control parameter in the EU [24,45], the dynamics were significantly different (ANOVA, *p* < 0.05; Table 2) among packaging forms (Figure 2c). Fresh fillets’ TVB-N content was 11.13 ± 0.01 (±SD) and decreased slightly to ca. 8–10 mg N/100 during the initial 24 h. Then, it increased steeply in fillets in AIR to >45 mg N/100 g just after 5 days of refrigerated storage, and then fluctuated between 25–35 mg N/100 g until the end of the storage trial. In the case of VP, fillets’ TVB-N content increased to >35 mg N/100 g on days 10 and 15 of storage. In contrast, the TVB-N content of fillets in MAP wavered below 25 mg N/100 g during the storage trial. Maximum limits for TVB-N content of 25–35 mg N/100 g, depending on fish species (but not triggerfish), are stipulated in EU Regulation 1022/2008 that amended Regulation 2074/2005 [24,45].

The TVB-N content in fresh fish is expectedly non-zero, since ammonia is a metabolite already present in the muscle. The concentration in fresh fillets was in line with TVB-N in fresh fillets of obscure pufferfish (ca. 7 mg N/100 g) [46], but way lower when compared to the initial value found by Pacheco-Aguillar et al. [12] for finescale triggerfish *B. polylepis* (ca. 25 mg N/100 g) and indicative of extremely fresh fish. Additionally, comparatively low concentrations were found in fresh fillets of other species, e.g., barramundi, <5 mg N/100 g [42], cultured sea bass (day 1), 7.34 mg N/100g [52], rainbow trout, ca. 10 mg N/100 g [49], meagre, 14.6 mg N/100 g [13], red mullet, 12.23 mg N/100g, and goldband goatfish, 19.49 mg N/100g [53]. Fuentes-Amayo et al. [54] found relatively higher values, 20–27 mg N/100 g, in the case of fresh barramundi, Atlantic salmon, blue-spotted emperor, saddletail snapper, and crimson snapper fillets after just one day of storage.

VP delayed and MAP inhibited the increase in TVB-N contents of gray triggerfish fillets compared to fillets packed in AIR. These differences are line with findings reported for other lean fish [13,42,43,52] and expected since modifying the headspace of packages delays bacterial activity and chemical reactions [20,55]. In contrast, the dynamics of TVB-N content in rainbow trout fillets in air, vacuum, and different atmospheres was matching and exceeded 25 mg N/100g after 10–12 days of chilled storage [56]. Cypian et al. [57] observed slow TVB-N accumulation (<25 mg N/100 g) during chilled and super-chilled storage of in air and modified atmosphere-packed tilapia fillets for 20 days.

### 3.2. Microbiological Parameters

**Figure 3:**
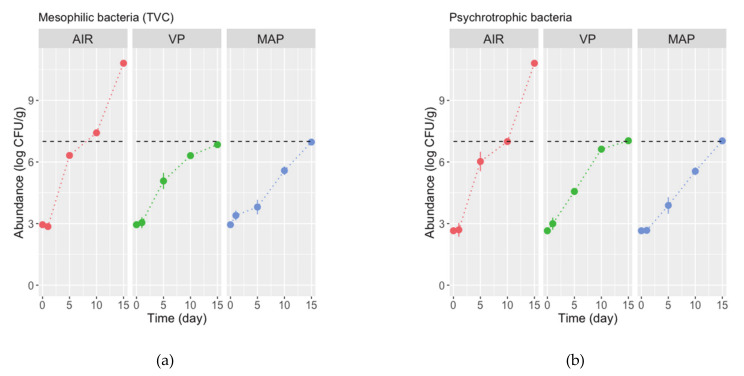

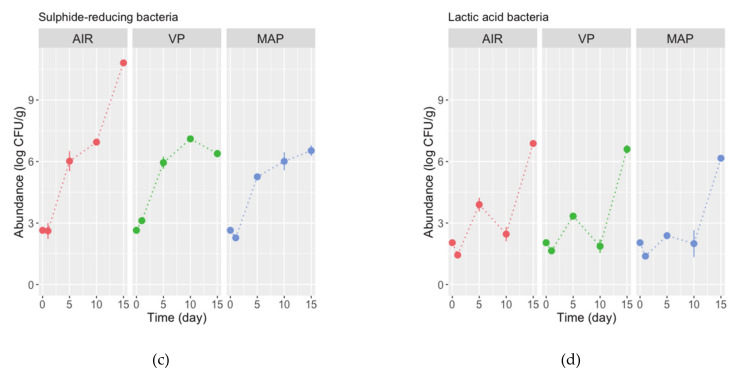
Changes in abundance of microbiota, (**a**) mesophilic, (**b**) psichrotrophic, (**c**) sulphide-reducing and (**d**) lactic acid bacteria, in fillets of triggerfish packed in air (AIR), vacuum (VP), and modified atmosphere (MAP) stored refrigerated for 15 days. Values are means of *n* = 2 fillets and whiskers represent the standard-error (SE). Horizontal dashed line in (**a**) represent the limit proposed by ICMSF [58].

In terms of microbiological quality and safety parameters, the abundance of total mesophiles (TVC) increased steadily and significantly (ANOVA, *p* < 0.05; Table 3) from 2.95 ± 0.02 log(CFU/g) in fresh fillets to >10 log(CFU/g) in fillets packed in AIR, and to ca. 6.8 log(CFU/g) in VP and MAP fillets at the end of the storage trial (day 15) (Figure 3a). A very similar pattern of change in and estimates of abundance was observed for psychrotrophic bacteria (Figure 3b) and sulphide-reducing bacteria (Figure 3c). Low initial abundances of sulphide-reducing bacteria indicate good hygienic conditions. Lactic acid bacteria (LAB) abundance in fresh fillets was low, 2.04 ± 0.03 log(CFU/g). Then, it fluctuated between 1.4 and 3.9 log(CFU/g) from day 1 to day 10, before increasing to 6.2–6.9 log(CFU/g) on day 15 (Figure 3d). TVC and psychrothropic bacteria of fillets in AIR, exceeded the proposed conventional limit of 7 log(CFU/g) by the International Commission on the Microbiological Specifications for Foods (ICMSF) [58] for foods on day 10, while the TVC and psychrotrophic bacteria in fillets packed in VP and MAP, despite increasing, remained below that limit during the trial.

Expectedly, VP and particularly MAP significantly retarded the growth of mesophilic and, more importantly, of psychrotrophic bacteria in triggerfish fillets stored refrigerated. Psychrotrophic bacteria are the main contributors to the spoilage of seafoods at refrigeration temperatures [59,60]. A similar retarding effect was observed by, for example, Arashisar et al. [56] in VP and MAP rainbow trout fillets, Masnyion et al. [42] in MAP seabass slices, Genç et al. [13] in VP meagre fillets, Giménez et al. [43] in VP/MAP trout fillets, and Kostaki et al. [52] in filleted seabass in MAP. Herein, a retarding effect was also observed for sulphide-reducing bacteria, but not for LAB. These bacteria showed a kind of “extended lag period” and increased sharply only after 10 days of storage. In fact, LAB dominated the final bacterial population in MAP gilthead seabream fillets [55]. LAB are resistant to CO_2_, and thus play a relevant role in the spoilage process of MAP finfish species, both fatty and lean species from warm waters [55,59,61]. VP/MAP has been shown to inhibit the normal spoilage microbiota in fishery products and substantially extend shelf-life, but rigorous temperature control is often necessary [20] and shelf-life extension depends on species and storage conditions [55], i.e., mixture of gases and temperature. According to Noseda et al. [59], “a change in atmosphere and in storage temperature from 0 °C (under ice) up to 4–7 °C (in MAP), implies a change in the microbiological spoilage of fishery products”. Several articles, some reporting tremendous increases in shelf-life, others describing little or no shelf-life extension, have been published. Often, a 30–60% extension of shelf-life is observed when using elevated levels of CO_2_ [20].

### 3.3. Sensory Analysis

**Figure 4:**
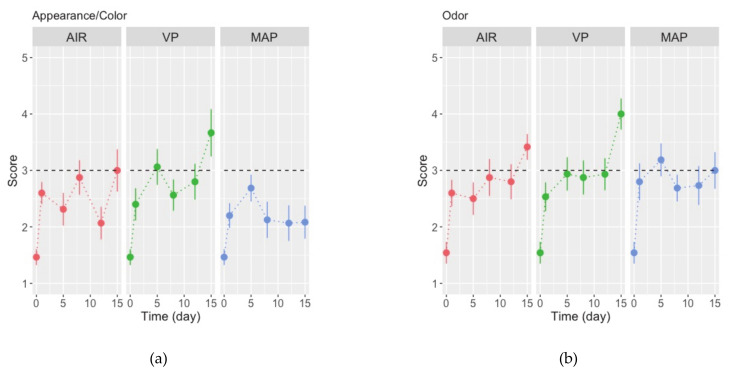

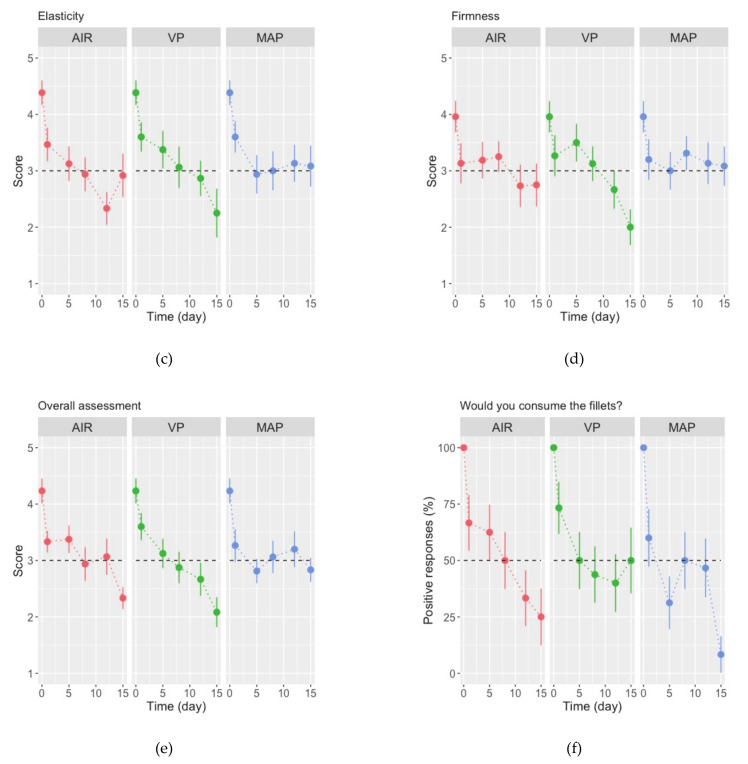
Changes in (**a**–**e**) sensory parameters and (f) in the intention to consume (%) fillets of triggerfish packed in air (AIR), vacuum (VP), and modified atmosphere (MAP) stored refrigerated for 15 days. Values are means of *n* = 14–16 panelists’ assessments, and whiskers represent the standard-error (SE). Horizontal dashed lines represent an intermediate score in the 1–5 scale in (**a**–**e**) and the 50% positive responses in (f).

The organoleptic attributes of triggerfish fillets perceived by a sensory panel changed clearly (Figure 4) and significantly (ANOVA, *p* < 0.05; Table 4) during the storage trial. In terms of appearance/color, fillets were perceived as translucent, shiny, sui generis, and pinkish for up to 15 days (mean scores ranged 1.5–3.1) in the case of AIR and MAP. In contrast, fillets in VP became dull, matte, and yellowish (this for some panelists) on day 15 (Figure 4a). Fresh fillets’ odor was classified as having fresh seaweed odor (scores of 1.5 ± 0.2). This changed abruptly to “neutral” or “little” odor after 24 h of storage (scores in the range of 2.5–2.8) in all fillets and remained as such until day 12 in fillets in MAP only. In the case of fillets in AIR and VP, fillets were perceived as “spoiled”, mean scores of 3.4 ± 0.2 and 4.0 ± 0.3, respectively, by the end of the trial (Figure 4b). The perception of texture of fillets (Figure 4c,d), in terms of elasticity and firmness, initially perceived as “elastic” and “firm” (scores of 4.0–4.4), decreased sharply during the initial 24 h (to 3.1–3.5), and then continued to decrease markedly in fillets in VP (to 2.0–2.2, slightly “elastic” and “soft”). Overall, fresh fillets were classified as “good” (mean scores of 4.2 ± 0.2). This initial impression decreased steeply to “reasonable” (scores of ca. 3.0) on day 12, and then to “bad” in the case of fillets in AIR and VP (Figure 4e).

The willingness to consume the fillets (%) decreased constantly with time of storage in fillets in AIR and MAP, reaching 50% on day 8. Conversely, ca. 50% of panelists were still willing to consume the fillets in VP after 15 days of chilled storage (Figure 4f).

Overall assessment scores of gray triggerfish fillets in AIR and VP were inversely related with color and odor changes (Figure 4a,b,e). Color is an important characteristic driving consumers’ decision on (sea)food products. In the case of MAP fillets’ texture attributes, elasticity and firmness were closely related with overall assessment (Figure 4c–e) and hence seemingly relevant. Masnyiom et al. [42] observed that a CO_2_-enriched atmosphere effectively extended the shelf-life of seabass slices by maintaining their odor and flavor attributes; however, it caused a change in sample color as well as exudate loss. Texture and exudate losses can be affected when using MAP [20,59]. Additionally, lower sensory evaluations of MAP fish can be found at terminal storage time due to LAB [61]. Furthermore, panelists rejected the samples (i.e., 50% positive responses or less for the intention to consume) when fillets were characterized as having no color (slightly translucent and shiny in MAP fillets) and neutral odor (odor of spoiled fish in VP) and being less elastic and softer (more so in VP). This occurred on day 8 for AIR, day 15 for VP, and day 12 for MAP fillets.

### 3.4. Shelf-Life Prediction

Considering primarily sensory (overall assessment and, particularly, intention of consumption), but also biochemical (TVB-N content) and microbiological parameters (TVC and psychrotrophic bacteria abundance), the shelf-life of gray triggerfish fillets under air (AIR) was 8 days. Vacuum-packaged (VP) and modified-atmosphere packaged (MAP) extended the shelf-life to 15 and 12 days, respectively.

The estimated shelf-lives in this study (in the range 8 to 15 days) are shorter than the time reported by Pacheco-Aguillar et al. [12] for finescale triggerfish stored at 0 °C, 20 days. They proposed that such a prolonged shelf-life could be explained by a combination of endogenous enzymes and microbiota poorly adapted to chilling temperatures ca. 0 °C. The specimens of gray triggerfish used in this study inhabit temperate waters, wherein naturally occurring microbiota are different from tropical areas surveyed by Pacheco-Aguillar et al. in the Gulf of Mexico. Moreover, the storage trials were carried under refrigeration (ca. 4 °C). This induces change(s) in the microbiological spoilage of fishery products. In this context, and compared to fillets in AIR, VP and MAP extended the shelf-life by 87.5% and 50%, respectively. This is in line with the range of 39%–102% that can be deduced from Noseda et al. [59] and the 30–60% extension of shelf-life referred to by Sivertsvik et al. [20] when using vacuum or elevated levels of CO_2_ at low temperatures. Moreover, the high initial microbiological and biochemical quality of fillets used herein benefited the use of MAP [20].

## 4. Conclusions

In a 15-days storage trial at refrigerating temperature, we assessed the effect of packaging gray triggerfish fillets in air (AIR), in vacuum (VP), and in modified atmosphere (MAP) on various quality parameters: Physicochemical, microbiological, and sensory.

Color of fillets changed appreciably when measured with a colorimeter. Fillets became lighter (increased L*) and yellower (b*>>0) with the time of storage. Distinct patterns were observed for pH among treatments and seemingly were somewhat related to changes observed in TVB-N content and the microbiota. Unexpectedly, the increasing trend observed in the texturometer-derived hardness of VP and MAP fillets contrasts the sensory assessment, wherein panelists perceived a clear softening of fillets during the storage trial. VP delayed and MAP inhibited the increase in TVB-N contents of gray triggerfish fillets compared to fillets packed in AIR. TVC and psychrothrophic bacteria of fillets in AIR exceeded the conventional limit of 7 log(CFU/g) on day 10, while in fillets packed in VP and MAP, their abundance remained below that limit during the trial. The organoleptic attributes of triggerfish fillets perceived by a sensory panel changed significantly in all treatments during the storage trial. Willingness to consume the fillets decreased constantly in AIR and MAP, but not in VP fillets. In view of sensory, biochemical, and microbiological parameters, the shelf-life of gray triggerfish fillets under air (AIR) was 8 days. VP and MAP extended the shelf-life to 15 and 12 days, respectively.

Future research regarding the VP and MAP of gray triggerfish fillets could involve the optimization of mixtures of gases use and/or the application of combined processes. The former would involve the testing of other percentages of CO_2_ and O_2_. While the latter could be the prior light salting or brining of fillets, the pre-treatments with preservatives, e.g., potassium sorbate or sodium acetate, or the application of essential (herbal) oils with bacteriostatic/antimicrobial and/or antioxidant properties. Additionally, incorporating/coating the packaging films with substances that inhibit or delay microbial, enzymatic, and/or oxidative spoilage of seafood by releasing the active agents in at a controlled rate to the inner environment or directly to the food (active packaging), could be examined to increase the shelf life. These hurdle technologies reinforce the effects of VP and MAP on food safety, and are expected to contribute to prolong the shelf-life even further.

## Figures and Tables

**Table 1:** Sensory parameters assessed in triggerfish fillets packed in air (AIR), vacuum (VP), and modified atmosphere (MAP).

Parameter	Description per Score				
	1	2	3	4	5
Appearance/Color	Translucent, shiny, *sui generis*, pink	Slightly translucent and shiny, slightly pink	No color	Dull, dimmed	Very dull, matte, yellowish, without shine
Odor	Fresh seaweed	Slightly fresh odor	Neutral, little odor	Spoiled fish	Putrid, ammoniacal
Elasticity ^1^	Not elastic				Very elastic
Firmness ^2^	Not firm, soft				Very firm, hard
Overall assessment	Very bad	Bad	Reasonable	Good	Very good

^1^ Rate of recovery after stopping the application of a force. ^2^ Force necessary to obtain a given deformation.

**Table 2:** Results of ANOVA per physicochemical quality parameter considering factors time (0, 1, 5, 10, and 15 days) and packaging (AIR, VP, and MAP).

Parameter	Factors	F	*p*-Value
L*	Time Packaging Time × Packaging	9.77 0.63 0.09	0.0036 0.5364 0.9135
a*	Time Packaging Time × Packaging	10.30 0.02 1.08	0.0029 0.9731 0.3497
b*	Time Packaging Time × Packaging	42.36 1.82 1.65	<0.0001 0.1770 0.2066
C	Time Packaging Time × Packaging	40.98 1.81 1.63	<0.0001 0.1789 0.2100
h*	Time Packaging Time × Packaging	29.90 0.51 0.81	<0.0001 0.8450 0.6720
ΔE	Time Packaging Time × Packaging	0.01 1.178 0.23	0.9045 0.3294 0.7970
pH	Time Packaging Time × Packaging	1.67 17.47 5.11	0.2039 <0.0001 0.0108
Hardness	Time Packaging Time × Packaging	12.11 47.56 14.66	<0.0001 <0.0001 <0.0001
TVB-N	Time Packaging Time × Packaging	61.51 0.01 6.91	<0.0001 0.9874 0.0007

**Table 3:** Results of ANOVA per microbiological quality parameter considering factors time (0, 1, 5, 10, and 15 days) and packaging (AIR, VP, and MAP).

Parameter	Factors	F	*p*-Value
TVC	Time Packaging Time × Packaging	37,026 140.3 247.9	<0.0001 <0.0001 <0.0001
Psychrotrophic bacteria	Time Packaging Time × Packaging	9586 9177 101.4	<0.0001 <0.0001 <0.0001
Sulphide-reducing bacteria	Time Packaging Time × Packaging	38,858 3.37 57.23	<0.0001 0.0619 <0.0001
Lactic-acid bacteria	Time Packaging Time × Packaging	1351 1.67 4.25	<0.0001 0.2209 0.0077

**Table 4:** Results of ANOVA per sensory parameter considering factors time (0, 1, 5, 8, 12, and 15 days) and packaging (AIR, VP, and MAP).

Parameter	Factors	F	*p*-Value
Appearance/Color	Time Packaging Time x Packaging	9.13 5.64 1.64	<0.0001 0.0040 0.0959
Odor	Time Packaging Time x Packaging	13.63 0.63 0.67	<0.0001 0.5351 0.6125
Elasticity	Time Packaging Time x Packaging	10.98 0.38 0.72	<0.0001 0.6822 0.7063
Firmness	Time Packaging Time x Packaging	5.51 0.50 0.69	<0.0001 0.6047 0.7372
Overall assessment	Time Packaging Time x Packaging	15.66 0.47 0.88	<0.0001 0.6281 0.5525

## Data Availability

The data presented in this study are available on request from the corresponding author.
